# Excessive daytime sleepiness is associated with impaired antibody response to influenza vaccination in older male adults

**DOI:** 10.3389/fcimb.2023.1229035

**Published:** 2023-12-12

**Authors:** Huy Quang Quach, Nathaniel D. Warner, Inna G. Ovsyannikova, Naima Covassin, Gregory A. Poland, Virend K. Somers, Richard B. Kennedy

**Affiliations:** ^1^ Mayo Clinic Vaccine Research Group, Mayo Clinic, Rochester, MN, United States; ^2^ Department of Quantitative Health Services, Mayo Clinic, Rochester, MN, United States; ^3^ Department of Cardiovascular Medicine, Mayo Clinic, Rochester, MN, United States

**Keywords:** influenza vaccine, hemagglutination inhibition, daytime sleepiness, older adults, antibody response

## Abstract

**Background:**

The reduced effectiveness of standard-dose influenza vaccines in persons ≥65 years of age led to the preferential recommendation to use high-dose (HDFlu) or MF59-adjuvanted (MF59Flu) vaccines for this age group. Sleep is an important modulator of immune responses to vaccines and poor sleep health is common in older adults. However, potential effects of poor sleep health on immune responses to influenza vaccination in older adults remain largely unknown.

**Methods:**

We conducted a cohort study of 210 healthy participants age ≥65 years, who received either seasonal high-dose (HDFlu) or MF59-adjuvanted (MF59Flu) influenza vaccine. We assessed sleep characteristics in this cohort by standardized questionnaires and measured the antibody titer against influenza A/H3N2 virus in serum of study participants by hemagglutination inhibition assay on the day of immunization and 28 days thereafter. We then assessed the association between sleep characteristics and antibody titers.

**Results:**

Our results demonstrated that male, but not female, study participants with excessive daytime sleepiness had an impaired influenza A/H3N2-specific antibody response at Day 28 post-vaccination. No other associations were found between antibody titer and other sleep characteristics, including sleep quality and obstructive sleep apnea.

**Conclusion:**

Our results provide an additional and easily measured variable explaining poor vaccine effectiveness in older adults. Our results support that gaining sufficient sleep is a simple non-vaccine interventional approach to improve influenza immune responses in older adults. Our findings extend the literature on the negative influence of excessive daytime sleepiness on immune responses to influenza vaccination in older male adults.

## 1 Introduction

Although all age groups are susceptible to influenza infection, persons ≥65 years of age are the most vulnerable to complications, accounting for 70% of hospitalizations and 85% of deaths associated with influenza (https://www.cdc.gov/flu/highrisk/65over.htm). This high burden is partly due to lower vaccine efficacy (Jefferson et al., 2005), which results from the progressive decline of both humoral and cellular immune responses in a phenomenon known as immunosenescence (McElhaney and Effros, 2009). To compensate for these reduced immune responses, high-dose (HDFlu) and MF59-adjuvanted (MF59Flu) inactivated influenza vaccines are recommended for this age group in the United States (Grohskopf et al., 2020), but influenza vaccine effectiveness remains suboptimal. While individuals ≥65 years of age currently comprise 10% of the global population in 2022, this is projected to increase to 16% in 2050 (United Nations Department of Economic and Social Affairs, Population Division, 2022); hence, understanding the mechanisms underlying the lower effectiveness of influenza vaccination in this age group is increasingly important. Besides vaccine approaches such as increasing the antigen dose in the vaccine formulation and changing the route of administration for influenza vaccines (Quach and Kennedy, 2022), exploring other physiological factors potentially enhancing immune responses to influenza vaccination as non-vaccine approaches is equally important.

While different aspects of sleep play an important role in immune responses to vaccination (Irwin, 2002; Bryant et al., 2004; Irwin, 2015; Castrucci, 2018; Besedovsky et al., 2019; Zimmermann and Curtis, 2019), conflicting results have been observed in regard to their influence on the immunogenicity of influenza vaccines. For example, healthy adults (18 - 55 years of age) who slept less than 7 hours per night had an increased likelihood of developing symptomatic illness following viral exposure (Cohen et al., 2009; Prather et al., 2015). In healthy young adults (18 - 25 years of age), short sleep duration on the two nights before influenza vaccination was associated with lower influenza antibody titers 1 and 4 months following vaccination independent of baseline influenza antibodies, age, and sex (Prather et al., 2021). Experimentally induced partial and total sleep deprivation at the time of influenza vaccination resulted in consistently reduced early antibody production in healthy young adults, thus suggesting an association between insufficient sleep and impaired influenza immune responses (Spiegel et al., 2002; Benedict et al., 2012). Clinical sleep disorders have also been linked to altered susceptibility to influenza and antibody response to influenza immunization. Healthy young adults reporting insomnia were found to have lower antibody titers than healthy good sleepers both before and after influenza vaccination, and both poor sleep quality and insomnia status were associated with antibody levels in multivariate analysis (Taylor et al., 2017). Risks of influenza infection and influenza-related complications were higher in individuals with obstructive sleep apnea (OSA) than in matched controls (Chen et al., 2021) and untreated or non-compliant OSA patients were more likely to be hospitalized due to influenza infection compared to patients adherent to OSA treatment (Mok et al., 2020). In contrast, there were no differences in the antibody response to influenza vaccination between treatment-naïve OSA individuals and non-OSA controls, nor were any objective sleep measures or daytime sleepiness associated with the immune response (Dopp et al., 2007).

Total sleep time in older adults is generally lower than in younger adults, typically less than 6 hours per night, while sleep efficiency declines (Foley et al., 1995; Ohayon et al., 2004; Mazzotti et al., 2014). Risks of OSA and poor sleep quality also increase with advancing age (Miner and Kryger, 2017). While disrupted sleep has been implicated in the impairment of immune responses in young adults, we are not aware of any relationship between sleep-related symptoms and immune responses to vaccination in older adults. We hypothesized that multiple aspects of sleep may significantly influence the immune responses to the influenza vaccines preferentially recommended in older adults age ≥65 in the United States (HDFlu and MF59Flu). To test this hypothesis, we assessed sleep characteristics in 210 study participants ≥65 years of age, who were immunized with either HDFlu or MF59Flu, and measured their influenza A/H3N2-specific antibody responses.

## 2 Methods

Some of the methods used in this study were similar or identical to the methods used in our previous publication (Haralambieva et al., 2022). The sleep questionnaires used in this study are widely used and well validated (Buysse et al., 1989; Johns, 1991; Chung et al., 2008).

### 2.1 Ethics statement

The Mayo Clinic Institutional Review Board reviewed and approved this study (IRB No. 17-010601). All study participants provided their written, informed consents at the time of enrollment.

### 2.2 Study participants

From August to December 2018, we recruited 250 otherwise healthy participants age ≥65 years, who received either seasonal HDFlu or MF59Flu vaccine and met inclusion criteria (see **Supplementary Materials** for more details of inclusion and exclusion criteria). Both HDFlu and MF59Flu vaccines used in this cohort study were trivalent inactivated vaccines, formulated from three viral strains: i) A/H1N1 (A/Michigan/45/2015/pdm09-like strain), ii) A/H3N2 (A/Singapore/INFIMH-16-0019/2016 strain), and iii) B lineage (Colorado/06/2017-like Victoria strain). During the period of this study, participants were followed up and excluded from data analysis if they were infected with or exhibited symptoms of influenza-like illness. Height and weight of participants were collected to calculate body mass index (BMI). One year after vaccination, four standardized sleep questionnaires were sent to all study participants. They were asked to complete the questionnaires according to their sleep and health status at the time of vaccination.

### 2.3 Sleep questionnaires

All participants self-assessed their sleep health by answering four sets of questionnaires, including the STOP scale (Chung et al., 2008), the STOP-BANG scale (Chung et al., 2008), the Epworth Sleepiness Scale (Johns, 1991), and the Pittsburgh Sleep Quality Index (Buysse et al., 1989). These questionnaires are described in detail in **Supplementary Material**.

### 2.4 Measurement of influenza A/H3N2-specific antibody titer

**Figure 1:**
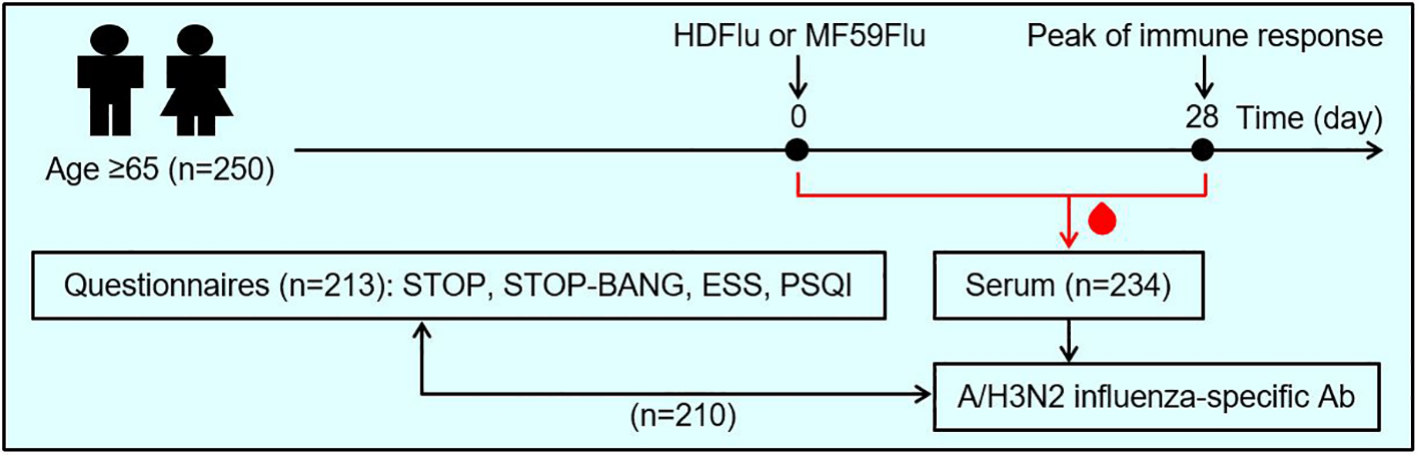
Study design. A total of 250 individuals ≥65 years of age enrolled for this study. These participants were asked to self-assess their sleep health by answering 4 sets of questionnaires, including: STOP, STOP-BANG (for the assessment of obstructive sleep apnea), the Epworth sleepiness scale (ESS, for the assessment of excessive daytime sleepiness), and the Pittsburgh sleep quality index (PSQI, for the assessment of sleep quality). The participants were randomized to receive either high-dose (HDFlu) or MF59-adjuvanted (MF59Flu) trivalent influenza vaccine. Blood was sampled from each participants before the time of vaccination (Day 0) and 28 days after immunization (Day 28) for the assessment of influenza A/H3N2-specific antibodies. Then, the effects of sleep characteristics, based on the outcome of 4 questionnaires, on antibody titer were evaluated. Of 250 enrolled individuals, 213 participants returned their completed questionnaires; 234 participants completed two visits for blood sampling at Day 0 and Day 28; 210 participants completed both questionnaires and blood sampling and were included in data analysis.

Blood was sampled from each study participant at two time points: before (Day 0) influenza vaccination and 28 days thereafter (Day 28), as summarized in **Figure 1**. Influenza A/H3N2-specific antibody titer was quantified by hemagglutination inhibition (HAI) assay and has been previously reported (Haralambieva et al., 2022). The coefficient of variation (CV) of the HAI assay was 2.9% (Haralambieva et al., 2022). Full details of blood collection and HAI assay are described in **Supplementary Material**.

### 2.5 Measurement of cytomegalovirus IgG

CMV IgG was quantified in serum samples using a commercially available ELISA kit (Bio-Rad, catalog no. 25177), following the manufacturer’s protocol. The concentration of CMV IgG was expressed as the sample index, which was calculated using the calibrator and the formula provided, as below.


$$Test\;sample\;index=\frac{calibrator\;index\times test\;sample\;absorbance}{calibrator\;absorbance}$$

According to the manufacturer, the assay has an average intra-assay coefficient of variation (CV) and inter-assay CV of 8.3% and 9.5%, respectively.

### 2.6 Statistical analysis

Pearson’s Chi-square test was used to compare differences between categorical variables. Wilcoxon ranked sum test was used to detect differences between continuous variables. Spearman’s correlation was used to determine the correlation between questionnaire scores and HAI antibody responses. A *p*<0.05 was used as a threshold for statistical significance. All statistical analyses were performed in RStudio (version 2022.02.3).

## 3 Results

### 3.1 Study participants had similar demographic and clinical characteristics

Demographic and clinical characteristics of the study cohort were briefly reported in our previous publication (Haralambieva et al., 2022). Of 250 individuals enrolled for this study, 210 participants completed both blood sampling and questionnaires and were included in data analysis (**Figure 1**; **Table 1**). Participants immunized with either HDFlu or MF59Flu vaccine were of similar ages with a median age of 71.1 and 71.5 years, respectively (*p*=0.782, **Table 1**). Participants also had comparable median BMI of 28.1 and 28.5 kg/m^2^ for HDFlu and MF59Flu vaccine groups, respectively (*p*=0.497, **Table 1**).

**Table 1:** Demographic and clinical characteristics of study participants (n = 210) and the score of their responses to questionnaires.

Characteristics	HDFlu (n = 103)	MF59Flu (n = 107)	*p* value
Age (year)			
All	71.1 (67.6, 76)	71.5 (68.2, 76.4)	0.7817
Female (n = 129)	70.7 (67.4, 77.1)	69.9 (66.9, 76.1)	0.5741
Male (n = 81)	71.4 (68.1, 75.6)	73.2 (70.0, 76.8)	0.1883
BMI (kg/m^2^)			
All	28.1 (25.0, 30.7)	28.5 (25.3, 31.7)	0.4970
Female	28.0 (23.8, 32.0)	28.4 (25.2, 31.6)	0.5694
Male	28.5 (25.9, 29.8)	28.7 (25.4, 31.7)	0.6891
STOP score			
All	1 (0, 2)	1 (0, 1)	0.3816
Female	1 (0, 2)	1 (0, 1)	0.7842
Male	1 (0.75, 2)	1 (0, 1)	0.3593
STOP-BANG score			
All	3 (2, 3)	2 (2, 3)	0.2742
Female	2 (1, 3)	2 (1, 3)	0.8487
Male	3 (2.75, 4)	3 (2, 3.75)	0.4812
ESS score			
All	4 (3, 7)	4 (2.5, 6.5)	0.7721
Female	4 (3, 6)	4 (2, 7)	0.7446
Male	4 (3, 8)	4 (3, 6)	0.4259
PSQI score			
All	5 (3, 7)	5 (3, 7)	0.7042
Female	5 (3, 8)	6 (3, 8)	0.6744
Male	4 (3, 6)	4 (2, 6.75)	0.6939
Log_2_(HAI) at Day 0			
All	5.32 (4.32, 6.32)	5.32 (4.32, 5.82)	0.6401
Female	5.32 (4.32, 6.32)	5.32 (4.32, 5.32)	0.1081
Male	5.32 (4.32, 6.32)	5.32 (4.32, 6.32)	0.1850
Log_2_(HAI) at Day 28			
All	6.32 (5.32, 7.32)	6.32 (5.32, 7.32)	0.1465
Female	6.32 (5.32, 7.32)	6.32 (5.32, 6.32)	0.0158
Male	6.32 (5.32, 7.32)	6.32 (6.32, 7.32)	0.3816

Data are presented as median (25^th^ quantile, 75^th^ quantile). Wilcoxon ranked sum test was used to assess the difference between two vaccine groups.

### 3.2 Nonsignificant association between sleep health and HAI titers

**Figure 2:**
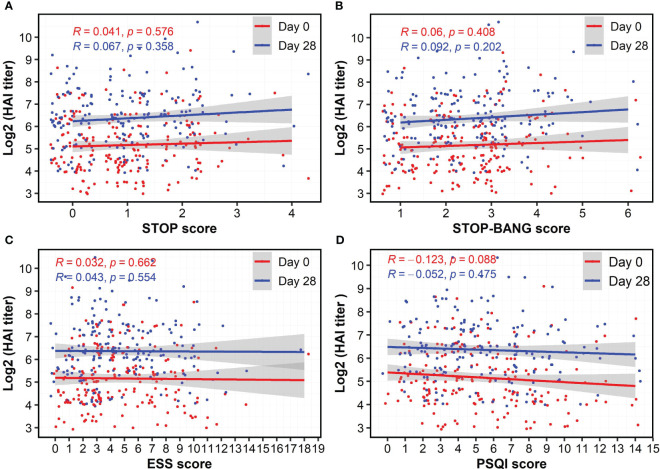
Nonsignificant correlations of HAI antibody titers at Day 28 and the score of questionnaires for obstructive sleep apnea **(A, B)**, excessive daytime sleepiness **(C)**, and sleep quality **(D)**. The HAI antibody titers were log-transformed. Spearman’s method was used to determine the correlation coefficient (R).

As a standard clinical laboratory-based method, the HAI assay was used to assess the presence of influenza A/H3N2-specific antibodies in serum samples. The two vaccine groups had the same median HAI titers at Day 0 and Day 28 (**Table 1**). We assessed a potential association between sex, vaccine type (HDFlu and MF59Flu), and the HAI titers. Using a linear regression model, we did not observe any significant influences of sex and vaccine type on the HAI titers after subtracting the HAI titer at baseline (Day 28-0) (**Supplementary Figures S1**, **S2**). Next, we explored whether the scores from each of the four questionnaires could be a predictor for the HAI titers. We found a nonsignificant association between the scores of STOP (for OSA), STOP-BANG (for OSA), and ESS (for excessive daytime sleepiness) questionnaires and the HAI titers at both Day 0 and Day 28 (**Figures 2A–C**), suggesting that these scores were not independent predictors of the HAI titers. Although the correlation coefficients indicated a negative impact of sleep quality (PSQI score) on the HAI titers, the correlation was not significant at both Day 0 (R=-0.123, *p*=0.088, **Figure 2D**) and at Day 28 (R=-0.052, *p*=0.475, **Figure 2D**).

CMV serostatus and BMI were also measured as potential factors that influence immune responses to influenza vaccinations in study participants. We found no significant correlations between these factors and the HAI titers (**Supplementary Figures S3**, **S4**), suggesting that these factors did not significantly influence the HAI titers.

### 3.3 Male participants with excessive daytime sleepiness had lower HAI titers

**Figure 3:**
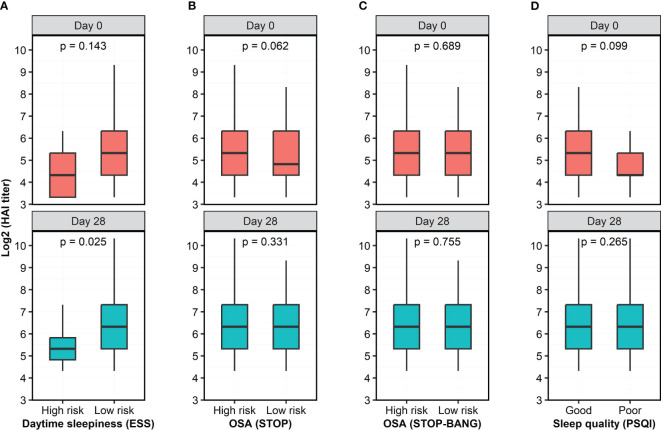
Antibody titers, as quantified by HAI assay, at two time points as a function of risk category of 4 questionnaires. **(A)** Participants with high risk of excessive daytime sleepiness had lower HAI titer than individuals with low risk of excessive daytime sleepiness. **(B, C)** There were no significant differences in HAI titers between participants with low and high risk of OSA, as measured by the STOP and STOP-BANG questionnaires. **(D)** Participants with poor and good sleep quality had similar HAI titers. The HAI titers were log-transformed while risk category was dichotomized based on the score of the questionnaires. Wilcoxon rank sum test with continuity correction was used to assess the difference in the HAI titer between dichotomized groups.

The standardized sleep questionnaires split individuals into low and high risk for each component of sleep characteristics, therefore we dichotomized study participants into low and high risk of OSA (**Supplementary Tables S1**, **S2**), without and with excessive daytime sleepiness (**Supplementary Table S3**), and poor and good sleep quality (**Supplementary Table S4**). At Day 0, participants with and without excessive daytime sleepiness had nonsignificantly different HAI titers (*p*=0.143, **Figure 3A**); however, the HAI titers measured in participants with excessive daytime sleepiness were significantly lower than in those without excessive daytime sleepiness at Day 28 (*p*=0.025, **Figure 3A**). Participants at low and high risk of OSA had similar HAI titers at Day 0 and Day 28 (**Figures 3B, C**). Participants with poor and good sleep quality also had comparable HAI titers at both Day 0 and Day 28 (**Figure 3D**). We also asked the participants to self-assess the presence of sleep apnea and did not observe a significant difference in the HAI titers between two groups who self-assessed “No” and “Yes” to sleep apnea (**Supplementary Figure S5**).

**Figure 4:**
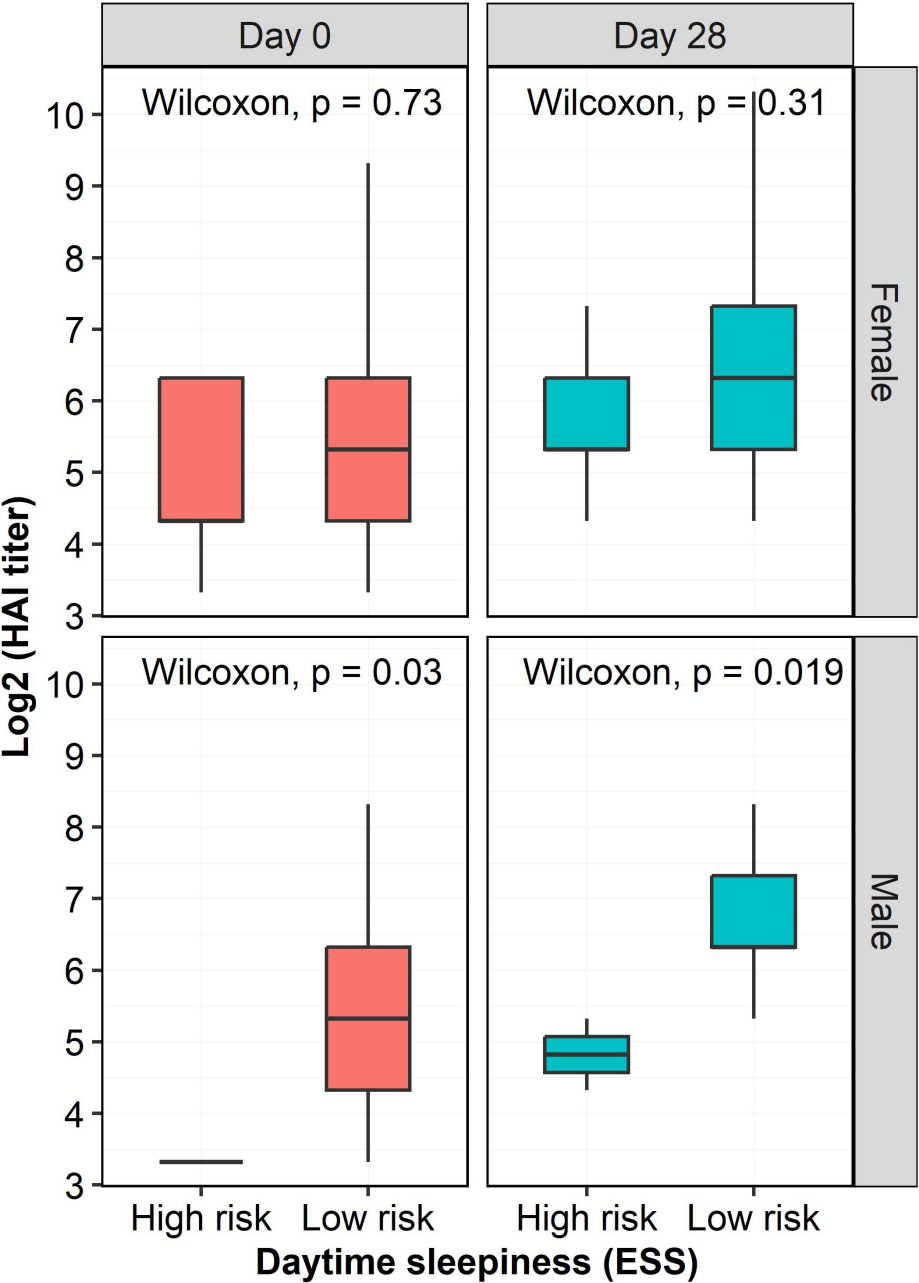
HAI titers at two time points as a function of risk of excessive daytime sleepiness and sex. HAI titers in males with a high risk of excessive daytime sleepiness were significantly lowers than those in males with a low risk of excessive daytime sleepiness at both Day 0 (*p*=0.03) and Day 28 (*p*=0.019). However, there were no significant influence of excessive daytime sleepiness on HAI titers in females. The HAI titers were log-transformed.

Since excessive daytime sleepiness had a negative impact on humoral responses after influenza immunization, we further explored whether the sex of the participants with excessive daytime sleepiness had a potential influence on HAI titers. We found that male, but not female, participants with excessive daytime sleepiness had significantly lower HAI titers at both Day 0 (*p*=0.03) and Day 28 (*p*=0.019) compared to male participants without excessive daytime sleepiness (**Figure 4**).

## 4 Discussion

Numerous vaccine approaches have been investigated to enhance the immunogenicity of influenza vaccines, but limited vaccine effectiveness was achieved in older adults (Quach and Kennedy, 2022); therefore, non-vaccine interventional approach that could improve immune responses to influenza vaccination in older adults should be alternatively explored. Meanwhile, a growing body of evidence suggests that sleep is an important immune modulator affecting immune response to influenza vaccination (Renegar et al., 1998; Spiegel et al., 2002; Prather et al., 2015; Castrucci, 2018; Prather et al., 2021). Since poor sleep is a common health issue in older adults, we explored potential effects of sleep-related symptoms on the humoral immune response to influenza vaccination. A key strength of our study lies in the study cohort of older adults, an understudied group who suffer from both low effectiveness of seasonal influenza vaccines and age-associated sleep issues. Overall, our findings suggest insufficient sleep as a non-vaccine factor contributing to poor vaccine effectiveness in older adults. The results from our study also provide evidence for a non-vaccine approach to improve the immunogenicity of influenza vaccines in older adults.

The interplay between sleep and the immune system is intricate (Irwin, 2002; Bryant et al., 2004; Irwin, 2015) and the mechanisms underlying the impact of inadequate sleep on inflammatory and antiviral responses have not been fully elucidated. Sleep disruption is thought to affect the regulation of immune system, primarily by altering activity of the hypothalamic–pituitary–adrenal axis and the sympathetic nervous system, which in turn results in potentiated transcription of proinflammatory genes and inhibited transcription of antiviral gene programs (Irwin, 2019). Acute sleep curtailment promotes activation of inflammatory signaling pathways and transcriptional signatures of inflammation, with a shift towards the production of T helper 2 cytokines and monocyte-derived IL-6 and TNF (Irwin et al., 2006; Irwin et al., 2015). Responses to vaccination are compromised by insufficient sleep, as demonstrated by decreased antibody production to influenza vaccination following total or partial sleep deprivation (Spiegel et al., 2002; Benedict et al., 2012; Prather et al., 2021). Conversely, evidence of impaired antibody production to influenza vaccination in individuals with overt sleep disorders is less solid (Dopp et al., 2007; Taylor et al., 2017). Notably, most of the data linking poor sleep to impaired vaccine response have been derived from studies on young or middle-aged adults (Spiegel et al., 2002; Cohen et al., 2009; Benedict et al., 2012; Prather et al., 2015; Taylor et al., 2017; Prather et al., 2021).

In the present work we observed, for the first time, significantly lower HAI titers post-vaccination in older adults reporting excessive daytime sleepiness compared to their non-sleepy counterparts (**Figure 3A**). Particularly, we found that excessive daytime sleepiness exerted a negative influence on humoral responses to influenza vaccines in males, but not females (**Figure 4**), which was consistent with results from a recent meta-analysis on numerous vaccines (Spiegel et al., 2023). While previous investigations have found that sleepiness is associated with increased levels of inflammatory markers (Vgontzas et al., 1997; Li et al., 2017) data on its implications in the context of immune response to influenza vaccination are scant. No association between sleepiness and antibody titers after influenza vaccination was observed in a group of middle-aged patients with sleep apnea, nor among young insomniacs (Dopp et al., 2007; Taylor et al., 2017). Taken together, these observations imply a pronounced negative effect of excessive daytime sleepiness on humoral responses to influenza vaccines in older male adults, but not in younger age groups. These observations also suggest that gaining sufficient sleep could be a simple non-vaccine approach to enhance the immunogenicity of influenza vaccines in older adults.

While the mechanistic link between daytime sleepiness and impaired immune response is not clear, it may be related to daily energy allocated for the immune system. As such, excessive daytime sleepiness likely reflects insufficient sleep, which is associated with impaired immune function (Watson et al., 2015). To function properly, the immune system requires a significant proportion of the daily energy budget; hence, our body needs to reduce the energy used for other activities by inducing sleep, saving energy for the immune system (Siegel, 2009). When sleep is insufficient, which consequently leads to excessive daytime sleepiness, adequate energy may not be available for proper immune function, resulting in impaired immune responses. Supporting this hypothesis, Lange et al. demonstrated that sufficient sleep enhanced the human antibody responses to hepatitis A vaccination by two-fold (Lange et al., 2003). Beyond immune responses, excessive daytime sleepiness has been associated with cognitive decline (Jaussent et al., 2012), increased cardiovascular mortality (Empana et al., 2009), and other adverse health outcomes in older adults (Bock et al., 2022). Our results indicate that the effect also exhibits a sex dependency. The possibility remains that the effect also occurs in females, but is not as pronounced as in males and we did not have a large enough cohort to detect it.

Using the STOP score as a measure, the risk of OSA in our study cohort was relatively high with 28.1% of participants having scores consistent with a high risk of OSA (**Supplementary Table S1**), as compared to 9 – 17% in 50-70-year-old individuals (Peppard et al., 2013). When BMI, age, neck size, and gender of participants were taken into consideration (STOP-BANG questionnaires), the risk of OSA was even higher with nearly 50% of participants at high risk of OSA (**Supplementary Table S2**). Meanwhile, 94.29% and 56.19% of participants reported no excessive daytime sleepiness and good sleep quality, respectively (**Supplementary Tables S3**, **S4**). As expected, a high proportion of subjects in the current study complained of poor sleep quality, making them an especially relevant cohort to explore the effects of sleep characteristics on immune responses to vaccination.

In our previous studies, demographic characteristics, including age and BMI, of study participants significantly affected the immune responses to influenza A/H1N1 vaccination in the elderly (Haralambieva et al., 2015; Voigt et al., 2019). The discrepancy in results may reflect that the current cohort received vaccines with superior immunogenicity and were able to ‘overcome’ the immunologic defects associated with age and/or obesity. Similar demographic characteristics in the two vaccine groups in this study minimized potential influences of such demographics on HAI titers.

We chose influenza A/H3N2 virus as a representative viral vaccine strain in the HAI assay because the hospitalization rate for A/H3N2 infection is significantly and demonstrably higher than for influenza A/H1N1 and type B infection (Reichert et al., 2004; Zhou et al., 2012) due to lower vaccine effectiveness against influenza A/H3N2 strain (Belongia et al., 2016). We found a negative impact of excessive daytime sleepiness (ESS score) on the generation of influenza A/H3N2-specific antibodies (**Figure 3A**), with a pronounced impact observed in males, but not females (**Figure 4**). Meanwhile, the HAI titers were not significantly different in participants having either a low or high risk of OSA, or poor or good sleep quality. Future studies designed to include individuals with more significant sleep disturbances would maximize the biologic differences and may avoid this bias.

There were several limitations in this pilot study. First, this is a convenience cohort recruited to study immune responses to influenza vaccination (Haralambieva et al., 2022). As participants were not initially recruited with the intention of studying sleep disturbances, the questionnaires were not answered at the time of immunization. Therefore, while we found a negative impact of excessive daytime sleepiness on the HAI titers, our results could be influenced by recall bias and the stability of sleep habits in our participants. In addition, the majority of study participants did not have excessive daytime sleepiness (**Supplementary Table S3**). A more comprehensive measure of sleep patterns in a larger cohort of participants intentionally recruited for a sleep study with a greater range of daytime sleepiness (and other sleep perturbations) is needed to confirm these insights. To this end, future studies should apply objective measurements of sleep and sleep symptoms, including overnight polysomnography and multiple sleep latency testing. Second, the data on HAI titers at different timepoints were limited in this study. A previous study found that sleep deprivation delayed the antibody responses in the early phase of 5-10 days after vaccination, but not during the late period of 2 weeks post-vaccination (Benedict et al., 2012). Future research should characterize the immune responses at different timepoints, including early phases of post-vaccination and long-term memory responses. Along similar lines, expanding the antibody testing to other influenza strains will allow investigators to determine if this effect broadly modulates the response to multiple virus strains or is specific to a given strain or set of antigens. Third, it is important to acknowledge that excessive sleepiness may also be secondary to medical or psychiatric conditions, such as major depression, or to medication use. Although our study population consisted of generally healthy participants, we cannot exclude that undiagnosed conditions may have confounded our results.

## 5 Conclusion

In conclusion, we investigated the effects of sleep on humoral immune responses to influenza HDFlu and MF59Flu vaccines recommended for older adults. Our results highlight a negative impact of excessive daytime sleepiness on humoral immune responses to influenza vaccination in older male adults. To the best of our knowledge, this study is the first study examining the association of daytime sleepiness on humoral immune responses to the two influenza vaccines recommended for older adults. These findings provide an additional and easily measured variable explaining poor vaccine effectiveness. Our findings support a hypothesis that gaining sufficient sleep is an alternative, non-vaccine, interventional approach to improve influenza immune responses in older adults.

## Data availability statement

The original contributions presented in the study are included in the article/**Supplementary Material**. Further inquiries can be directed to the corresponding author.

## Ethics statement

The studies involving humans were approved by The Mayo Clinic Institutional Review Board (IRB No. 17-010601). The studies were conducted in accordance with the local legislation and institutional requirements. The participants provided their written informed consent to participate in this study.

## Author contributions

VS, NC, GP, and RK were involved in the conceptualization of the study. RK, IO, and GP were involved in the funding acquisition and project administration. HQ analyzed data and wrote the first draft of the manuscript. NW was involved in data analysis. All authors were involved in the writing, editing, and revising of the manuscript. All authors contributed to the article and approved the submitted version.
