# Supplementary material for: Excessive daytime sleepiness is associated with impaired antibody response to influenza vaccination in older male adults
Source: Front Cell Infect Microbiol. 2023 Dec 12;13:1229035. doi: 10.3389/fcimb.2023.1229035 (PMC10749933; doi:10.3389/fcimb.2023.1229035)
Supplement: Supplementary file 1 [file DataSheet_1.pdf]

Supplementary Material

Excessive daytime sleepiness is associated with impaired antibody response to influenza vaccination in older male adults

Huy Quang Quach[1], Nathaniel D. Warner[2], Inna G. Ovsyannikova[2], Naima Covassin[3], Gregory A. Poland[1], Virend K. Somers[3], Richard B. Kennedy[1]

[1]Mayo Clinic Vaccine Research Group, Mayo Clinic, Rochester, MN 55905, USA

[2]Department of Quantitative Health Services, Mayo Clinic, Rochester, MN 55905, USA

[3]Department of Cardiovascular Medicine, Mayo Clinic, Rochester, MN 55905, USA

Running title: sleep's impact on responses to influenza vaccine

Keywords: influenza vaccine, hemagglutination inhibition, daytime sleepiness, older adults, antibody response

Correspondence address: kennedy.rick@mayo.edu (Richard B. Kennedy)

A. Supplementary methods

A.1. STOP questionnaire

The STOP questionnaire is an easy-to-use tool to screen obstructive sleep apnea (OSA) (Chung et al., 2008). It consists of 4 yes/no questions related to snoring, tiredness during daytime, observed apnea, and high blood pressure. Those who answered "yes" to <2 questions were classified as low risk of OSA. Otherwise, those who answered "yes" to ≥2 questions were classified as high risk of OSA. The score of the STOP questionnaire from all participants is summarized in Supplementary Table S1.

A.2. STOP-BANG questionnaire

Similar to STOP questionnaire, the STOP-BANG questionnaire has 4 additional yes/no questions related to body mass index (BMI), age, neck size, and gender (Chung et al., 2008). Those who answered "yes" to <3 and ≥3 questions were classified as low and high risk of OSA, respectively. The score of the STOP-BANG questionnaire from all participants is summarized in Supplementary Table S2.

A.3. Epworth Sleepiness Scale (ESS)

The ESS is routinely used to assess daytime sleepiness (Johns, 1991). The scale has 8 self-rated questions, each of which is rated on a 4-point Likert scales (0-3). An ESS score ≥11 was used to identify excessive daytime sleepiness. The score of the ESS questionnaire from all participants is summarized in Supplementary Table S3.

A.4. Pittsburgh Sleep Quality Index (PSQI)

The PSQI is used to assess sleep quality (Buysse et al., 1989). It contains a total of 24 questions, 19 of which are self-rated by study participants and 5 other questions are rated by their bed partner

or roommate (if available). Good and poor sleep quality were classified as PSQI scores of <6 and ≥6, respectively. The score of the PSQI questionnaire from all participants is summarized in Supplementary Table S4.

A.5. Hemagglutination inhibition (HAI) assay

Blood was sampled from each participant at two time points: Day 0 (before immunization) and Day 28 post-vaccination (peak of IgG responses to influenza vaccination) (Figure 1) (Gross et al., 1996). Blood samples were collected in BD Vacutainer® tubes containing a clot activator and left to clot for 30 min at room temperature. Clotted blood was centrifuged at 2000×*g* for 15 min at room temperature. Serum was collected in the supernatant after centrifugation and stored at -80°C until use.

Influenza-specific antibodies were quantified by hemagglutination inhibition (HAI) assay and generally reported in our previous publication. Briefly, influenza-specific antibodies were measured by hemagglutination inhibition (HAI) assay against A/H3N2 influenza virus (A/Singapore/INFIMH-16-0019/2016 strain), following WHO's guideline (Webster et al., 2002) and a previously optimized protocol (Kaufmann et al., 2017). Serum was first treated with receptor-destroying enzyme (RDE) (Sigma-Aldrich, St. Louis, MO) to eliminate non-specific inhibitors of hemagglutination. Treated serum was two-fold serially diluted with PBS in 96-well plate, starting from 1:10 dilution. Diluted serum (25 μL) was mixed with 25 μL of standardized H3N2 virus containing 4 hemagglutination units and incubated for 30 min at room temperature. Then, 50 μL of 0.5% turkey red blood cell suspension was added to the mixture of serum-incubated virus. Hemagglutination or inhibition thereof was read after incubation for 45 min. HAI titer was determined as the reciprocal of the highest serum dilution that completely inhibits hemagglutination.

A.6. Inclusion Criteria

To be included for further data analysis, all enrolled participants need to meet these inclusion criteria:

i. Male or female adults ages and older at the time of enrollment
ii. Eligible to receive Fluad® (MF59Flu) or Fluzone® (HDFlu) if age 65 or older
iii. No history of anaphylactic reaction to gelatin, neomycin, or other vaccine component
iv. No immunosuppression or immunodeficiency
v. No acute illness at time of vaccination
vi. Determined by medical history and clinical judgment to be eligible for the study, by being generally healthy, with no autoimmune or immunosuppressive conditions and having stable current medical conditions (subjects with preexisting stable disease, defined as disease not requiring significant change in therapy or hospitalization for worsening disease 12 weeks before receipt of study vaccine, will be eligible. A change in dose or therapy within a category (e.g., change from one nonsteroidal anti-inflammatory drug to another) is allowed. A change to a new therapy category (e.g., surgery or addition of a new pharmacological class) is only allowed if it is not caused by worsening disease. A change to a new therapy category caused by worsening disease is considered significant and therefore ineligible for enrollment.
vii. Patients with diabetes mellitus are eligible for inclusion if they have had a hemoglobin A1c measurement of <8.0 within the past 6 months prior to enrollment. These hemoglobin A1c measurements are recommended at least twice yearly by the American Diabetes Association (ADA), and the target levels here are representative of the goals of the ADA. These hemoglobin A1c levels will ensure that these participants have good glycemic

control. (American Diabetes Association. American Diabetes Association Position Statement: Standards of Medical Care in Diabetes— 2015. Diabetes Care 2015;38(Suppl. 1): S1–S94).

viii. Able to follow study procedures in the opinion of the investigator

ix. Expected to be available for the duration of the study

x. Weighs >110 lbs

A.7. Exclusion Criteria

Enrolled participants are excluded from data analysis if they meet one of the following exclusion criteria:

i. Known or suspected immunodeficiency or receiving treatment with immunosuppressive therapy including cytotoxic agents (e.g., for cancer, HIV, or autoimmune disease).

ii. Subjects on corticosteroids will be excluded if ≥ 20mg of Prednisone (or equivalent drug) has been (or will be) administered daily for 2 weeks or more. Subjects will be eligible if corticosteroid therapy has been discontinued for at least 30 days.

iii. Serious chronic medical conditions including metastatic malignancy, severe chronic obstructive pulmonary disease requiring supplemental oxygen, end-stage renal disease with or without dialysis, clinically unstable cardiac disease, or any other disorder that, in the investigator's opinion, precludes the subject from participating in the study. Diabetic patients will be excluded if they do not have a hemoglobin A1c measurement within the past 6 months or if they had a hemoglobin A1c measurement of an A1c >8.0.

iv. Receipt of any blood products, including immunoglobulin, within 6 months of study enrollment.

v. Current anticoagulant therapy or a history of bleeding diathesis that would contraindicate intramuscular (IM) injection. (Note: antiplatelet drugs such as aspirin and clopidogrel are permitted).

vi. Receipt of any vaccines within the past 30 days prior to enrollment.

vii. Receipt of the current seasonal influenza vaccine other than in this study.

viii. Acute illness within the last 30 days.

ix. Blood donation within the last 56 days prior to study enrollment and within 56 days following the last study visit.

x. Pregnancy, nursing or trying to conceive at the time of the study or for 28 days following the baseline visit.

xi. Any condition (e.g. allergic reaction, Guillain-Barre Syndrome) that precludes their receipt of the influenza vaccine

xii. Currently taking antibiotics to treat a serious infection. Preventative use of antibiotics (i.e. oral surgery) is not an exclusion criterion.

xiii. Diagnosis of a cognitive disorder (e.g. Alzheimer's, Dementia)

xiv. Anemia

xv. Any medical conditions that would, in the opinion of the investigator, interfere with the evaluation of the study objectives.

B. Supplementary data

Supplementary Table S1. Summary of STOP score.

STOP questionnaire	Frequency (%)
**Raw score**	
0	73 (34.76%)
1	74 (35.24%)
2	47 (22.38%)
3	8 (3.81%)
4	4 (1.9%)
Not available	4 (1.9%)
**STOP risk category**	
Low risk (score <2)	147 (70%)
High risk (score ≥2)	59 (28.1%)

Supplementary Table S2. Summary of STOP-BANG score.

STOP-BANG questionnaire	Frequency (%)
**Raw score**	
1	47 (22.38%)
2	56 (26.67%)
3	70 (33.33%)
4	22 (10.48%)
5	8 (3.81%)
6	4 (1.9%)
Not available	3 (1.43%)
**STOP-BANG risk category**	
Low risk (score <3)	103 (49.05%)
High risk (score ≥3)	104 (49.52%)

Supplementary Table S3. Summary of likelihood of daytime sleepiness (ESS) score.

ESS questionnaire	Frequency (%)
**Raw score**	
0	10 (4.76%)
1	18 (8.57%)
2	24 (11.43%)
3	40 (19.05%)
4	20 (9.52%)
5	19 (9.05%)
6	18 (8.57%)
7	18 (8.57%)
8	10 (4.76%)
9	12 (5.71%)
10	9 (4.29%)
11	3 (1.43%)
12	4 (1.9%)
14	1 (0.48%)
18	1 (0.48%)
Not available	3 (1.43%)
**ESS risk category**	
No excessive daytime sleepiness (score <11)	198 (94.29%)
Excessive daytime sleepiness (score ≥11)	9 (4.29%)

Supplementary Table S4. Summary of PSQI score.

PSQI questionnaire	Frequency (%)
**Raw score**	
0	2 (0.95%)
1	17 (8.1%)
2	20 (9.52%)
3	25 (11.9%)
4	31 (14.76%)
5	23 (10.95%)
6	18 (8.57%)
7	24 (11.43%)
8	15 (7.14%)
9	9 (4.29%)
10	8 (3.81%)
11	5 (2.38%)
12	5 (2.38%)
14	4 (1.9%)
Not available	4 (1.9%)
**PSQI risk category**	
Good sleep quality (score <6)	118 (56.19%)
Poor sleep quality (score ≥6)	88 (41.9%)

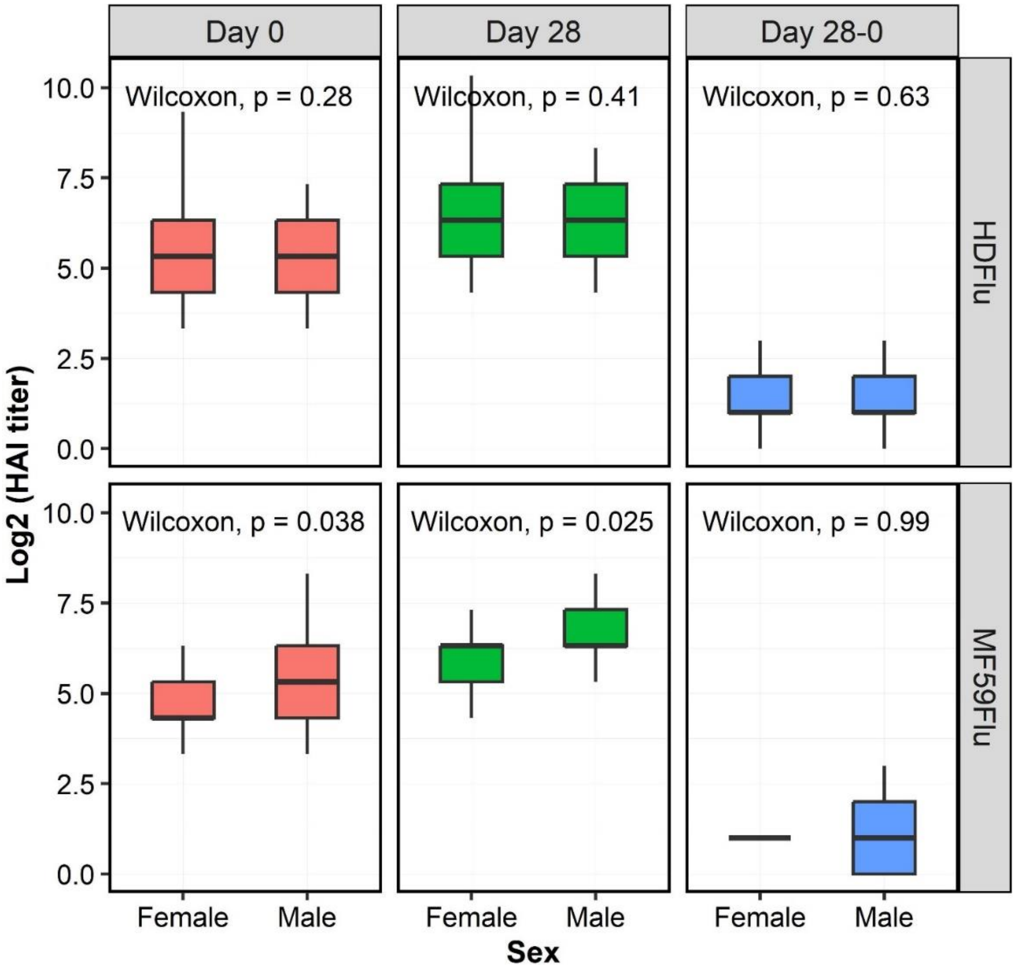

Supplementary Figure S1. HAI titers at two time points as a function of vaccine and sex. Results in "Day 28-0" panel are the change in HAI titers between 2 time points (HAI titer at Day 28 - HAI titer at Day 0). The HAI titers were log-transformed. Wilcoxon test was used to assess the difference in HAI titer between females and males. Although there were significant differences in HAI titer between female and male, who received MF59Flu, at both Day 0 (p=0.038) and Day 28 (p=0.025), the difference was no longer significant (p=0.99) after subtracting the baseline HAI titer (Day 28-0).

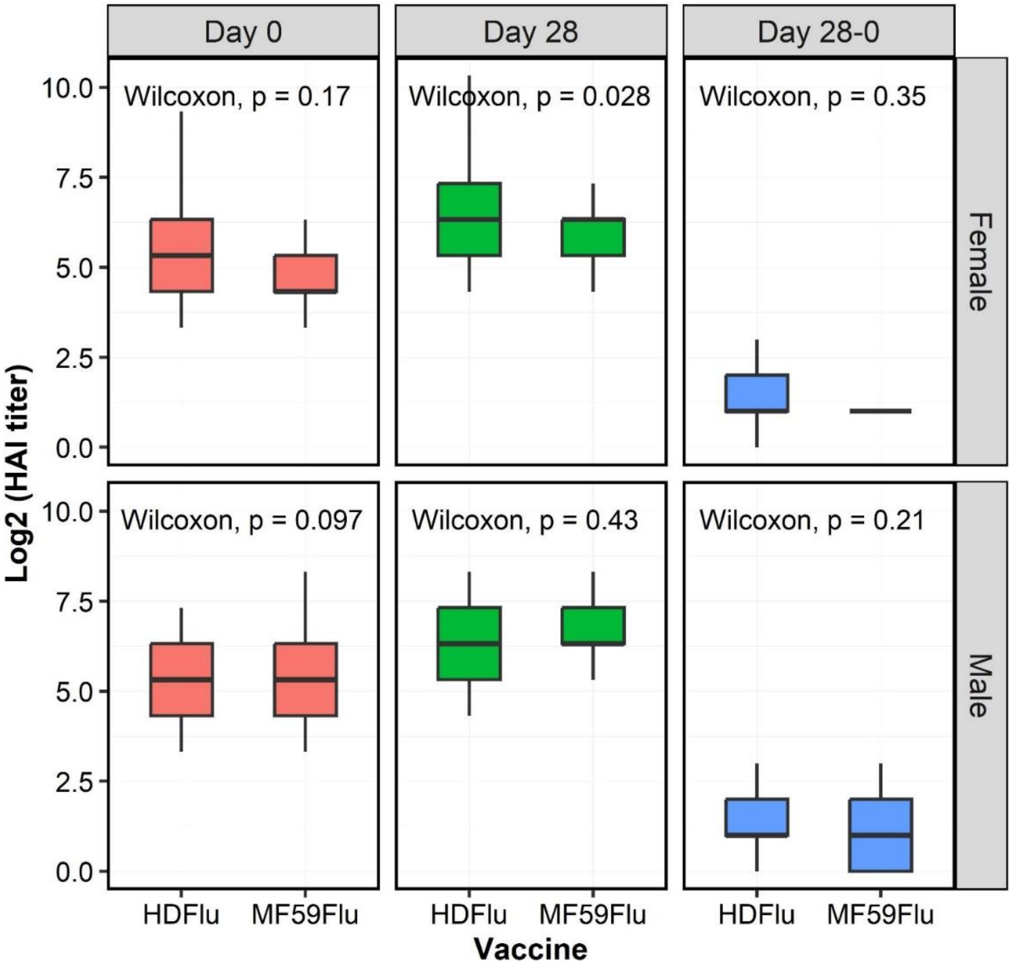

Supplementary Figure S2. HAI titers at two time points as a function of sex and vaccine. Results in "Day 28-0" panel are the change in HAI titers between 2 time points (HAI titer at Day 28 - HAI titer at Day 0). The HAI titers were log-transformed. Wilcoxon test was used to assess the difference in HAI titer between two vaccines. Although there was a significant difference in HAI titer between females of HDFlu and MF59Flu groups at Day 28 (p=0.028), the difference was not significant (p=0.35) after subtracting the baseline HAI titer (Day 28-0).

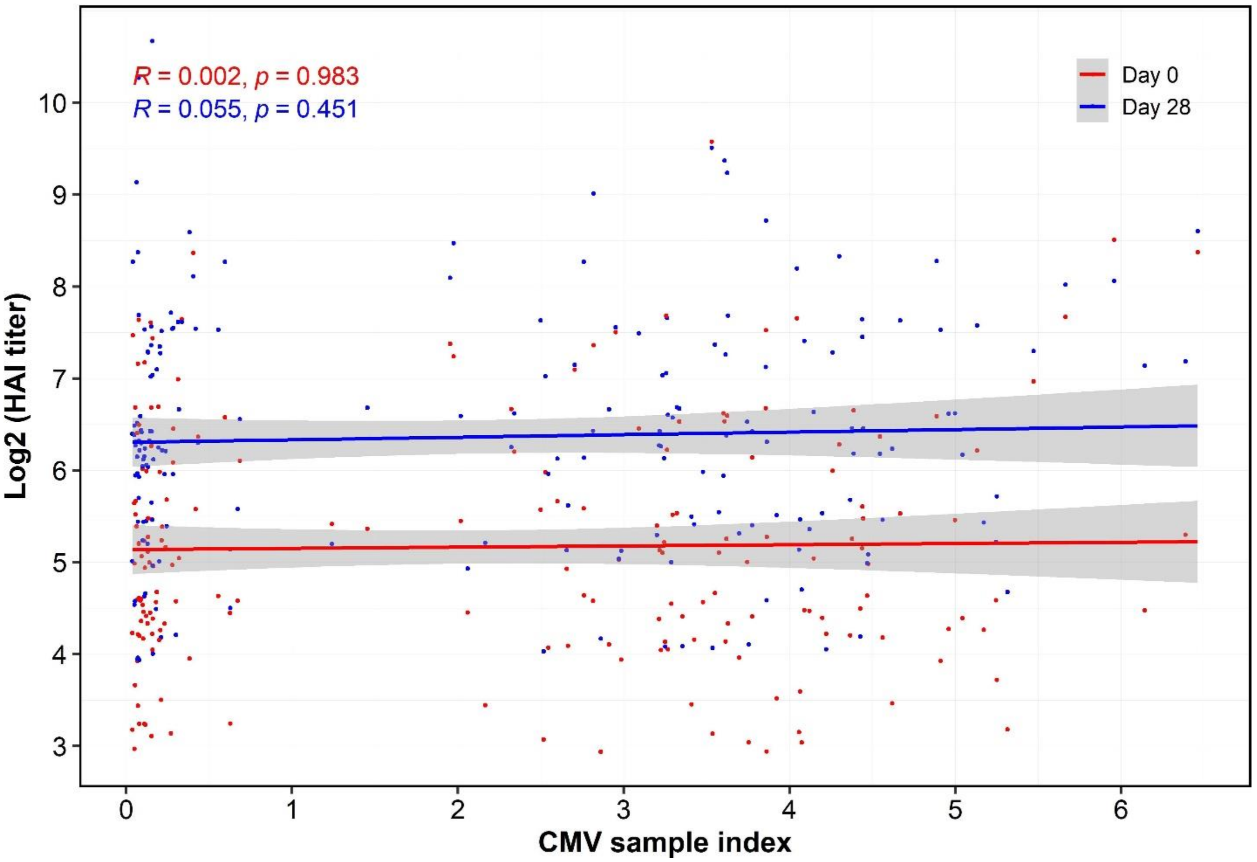

Supplementary Figure S3. Nonsignificant correlation between CMV IgG sample index and HAI titer. Spearman method was used to assess the correlation between CMV IgG sample index and HAI titer at both Day 0 and Day 28.

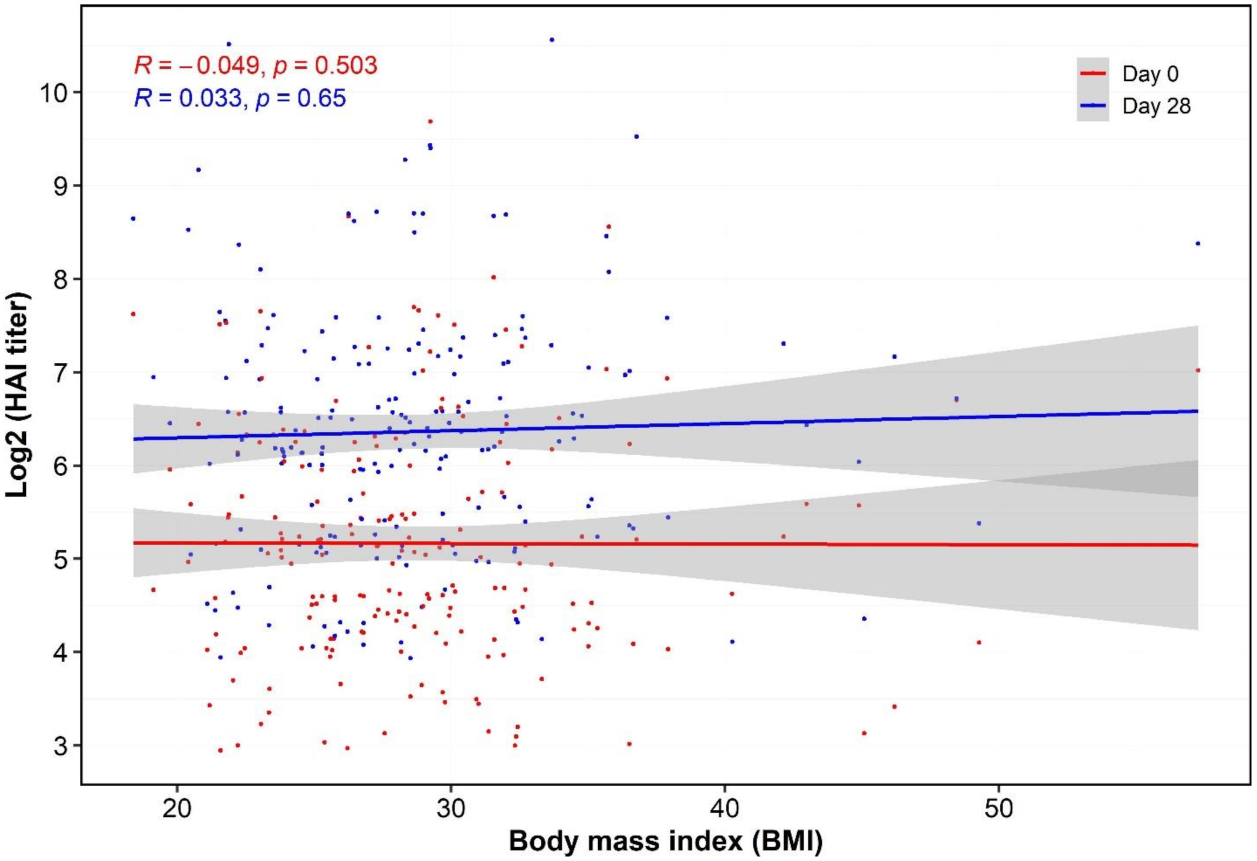

Supplementary Figure S4. Nonsignificant correlation between BMI and HAI titer. Spearman method was used to assess the correlation between BMI and HAI titer at both Day 0 and Day 28.

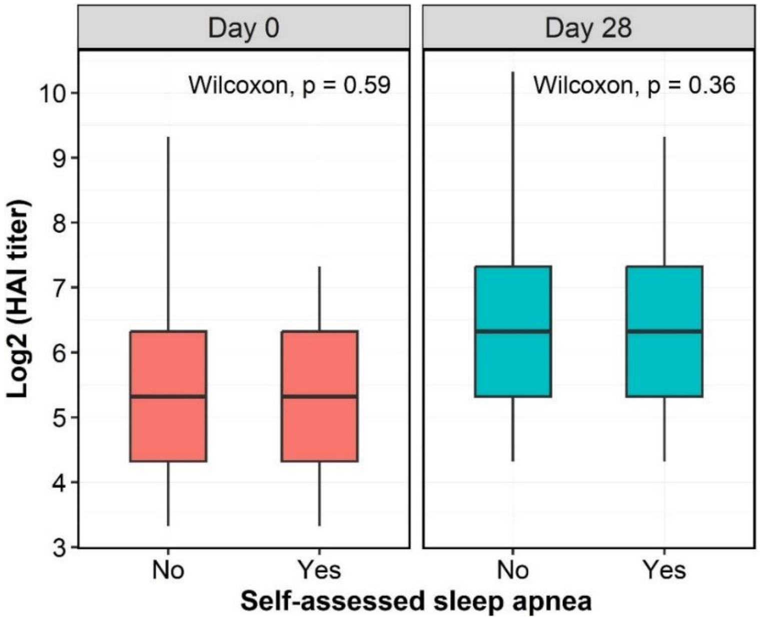

Supplementary Figure S5. Nonsignificant difference in the HAI titer in participants who self-assessed "No" and "Yes" to sleep apnea. The HAI titers were log-transformed. Wilcoxon test was used to assess the difference in HAI titer between two groups.

References

Buysse, D.J., Reynolds, C.F., 3rd, Monk, T.H., Berman, S.R., and Kupfer, D.J. (1989). The Pittsburgh Sleep Quality Index: a new instrument for psychiatric practice and research. *Psychiatry Res* 28**,** 193-213.

Chung, F., Yegneswaran, B., Liao, P., Chung, S.A., Vairavanathan, S., Islam, S., Khajehdehi, A., and Shapiro, C.M. (2008). STOP questionnaire: a tool to screen patients for obstructive sleep apnea. *Anesthesiology* 108**,** 812-821.

Gross, P.A., Russo, C., Teplitzky, M., Dran, S., Cataruozolo, P., and Munk, G. (1996). Time to peak serum antibody response to influenza vaccine in the elderly. *Clin Diagn Lab Immunol* 3**,** 361-362.

Johns, M.W. (1991). A new method for measuring daytime sleepiness: the Epworth sleepiness scale. *Sleep* 14**,** 540-545.

Kaufmann, L., Syedbasha, M., Vogt, D., Hollenstein, Y., Hartmann, J., Linnik, J.E., and Egli, A. (2017). An optimized hemagglutination inhibition (HI) assay to quantify influenza-specific antibody titers. *Journal of visualized experiments: JoVE*.

Webster, R., Cox, N., and Stöhr, K. (2002). "WHO Animal Influenza Manual". WHO/CDS/CSR/NCS).
